# The comparison of Proseal laryngeal mask airway and endotracheal tube in patients undergoing laparoscopic surgeries under general anaesthesia

**DOI:** 10.4103/0019-5049.79891

**Published:** 2011

**Authors:** Namita Saraswat, Aditya Kumar, Abhijeet Mishra, Amrita Gupta, Gyan Saurabh, Uma Srivastava

**Affiliations:** Department of Anaesthesia, Lady Hardinge Medical College, New Delhi, India; 1 Department of Anaesthesia, S.N. Medical College, Agra, India; 2 Department of Anaesthesia, RML Hospital, New Delhi, India, India; 3 Department of Surgery, Lady Hardinge Medical College, New Delhi, India

**Keywords:** Endotracheal tube, IPPV, laparoscopy, oropharyngeal seal pressure, Proseal LMA

## Abstract

Aims to compare the efficacy of Proseal laryngeal mask airway(PLMA) and endotracheal tube (ETT) in patients undergoing laparoscopic surgeries under general anaesthesia. This prospective randomised study was conducted on 60 adult patients, 30 each in two groups, of ASA I-II who were posted for laparoscopic procedures under general anaesthesia. After preoxygenation, anaesthesia was induced with propofol, fentanyl and vecuronium. PLMA or ETT was inserted and cuff inflated. Nasogastric tube (NGT) was passed in all patients. Anaesthesia was maintained with N_2_ O, O_2_, halothane and vecuronium. Ventilation was set at 8 ml/kg and respiratory rate of 12/min. The attempts and time taken for insertion of devices, haemodynamic changes, oxygenation, ventilation and intraoperative and postoperative laryngopharyngeal morbidity (LPM) were noted. There was no failed insertion of devices. Time taken for successful passage of NGT was 9.77 s (6-16 s) and 11.5 s (8-17 s) for groups P and E, respectively. There were no statistically significant differences in oxygen saturation (SpO_2_) or end-tidal carbon dioxide (EtCO_2_) between the two groups before or during peritoneal insufflation. Median (range) airway pressure at which oropharyngeal leak occurred during the leak test with PLMA was 35 (24-40) cm of H_2_O. There was no case of inadequate ventilation, regurgitation, or aspiration recorded. No significant difference in laryngopharyngeal morbidity was noted. A properly positionedPLMA proved to be a suitable and safe alternative to ETT for airway management in elective fasted, adult patients undergoing laparoscopic surgeries. It provided equally effective pulmonary ventilation despite high airway pressures without gastric distention, regurgitation, and aspiration.

## INTRODUCTION

In spite of tremendous advances in contemporary anaesthetic practice, advances, airway management continues to be of paramount importance to anaesthesiologists. Till date, the cuffed tracheal tube was considered as the gold standard for providing a safe glottic seal, especially for laparoscopic procedures under general anaesthesia.[1] The disadvantages of tracheal intubation, which involves rigid laryngoscopy, are in terms of concomitant haemodynamic responses and damage to the oropharyngeal structures at insertion. Postoperative sore throat is also a serious concern. This precludes the global utility of the tracheal tube and requires a better alternative.[2] Over a period of time, new airway devices have been added to the anaesthesiologist’s armamentarium.

Proseal laryngeal mask airway (PLMA) has a dorsal cuff, in addition to the peripheral cuff of LMA, which pushes the mask anterior to provide a better seal around the glottic aperture and permits high airway pressures without leak. The drain tube parallel to the ventilation tube permits drainage of passively regurgitated gastric fluid away from the airway and serves as a passage for gastric tube.[2] The PLMA is a relatively new airway device in developing nations. This study is therefore undertaken to compare PLMA with standard tracheal tube for the number of attempts and time taken for insertion, haemodynamic changes, oxygenation, ventilation and intraoperative and postoperative laryngopharyngeal morbidity (LPM) occurring during general anaesthesia in young healthy adult patients undergoing laparoscopic surgeries.

## METHODS

After obtaining the Ethics committee approval and written informed consent, this prospective randomised study was conducted on 60 healthy patients. The patients were of either sex belonging to ASA physical status grade I and II, aged 20-65 years and body weight 40-76 kg, who underwent laparoscopic procedures under general anaesthesia. Patients with anticipated difficult airway, obesity (body mass index > 35 kg/m^2^), oropharyngeal pathology, cardiopulmonary disease, cervical spine fracture or instability, or at increased risk of aspiration (gastro-esophageal reflux disease, hiatus hernia, and pregnant patients) were excluded from the study.

Patients were randomised for airway management with the PLMA or endotracheal tube (ETT) by opening an opaque envelope inside the operation theatre containing the computer-generated random assignment into two groups of 30 each. Patients in group P were to receive a PLMA and patients in group E were to undergo endotracheal intubation. Patients were premedicated with oral alprazolam 0.5 mg the night before surgery and on the day of surgery. After intravenous (IV) access was obtained, ranitidine 50 mg and metoclopramide 10 mg were administered 30 minutes before surgery. In the operation theatre, standard monitors were attachedand baseline parameters were recorded. Injections of midazolam 0.02 mg/kg, glycopyrrolate 0.005 mg/kg, and fentanyl 1-2 μg/kg were administered 1-2 min before induction. After preoxygenation with 100% O_2_ for 3-5 minutes, anaesthesia was induced with injection of propofol 2-2.5 mg/kg till the loss of verbal commands. Neuromuscular blockade to facilitate placement of device was achieved by vecuronium 0.08-0.1 mg/kg. Following induction and adequate paralysis, the corresponding airway was inserted in each group. The airway devices were inserted by anaesthesiologists with at least 1 year experience with PLMA and ETT. In group P, size 3 or 4 PLMA (according to weight) was used. For the purpose of standardisation, we used the introducer for inserting the PLMA for all cases as recommended by the manufacturer. In group E, endotracheal intubation (7.5 in females and 8 in males) was performed in standard manner. The time interval between holding the airway device to confirmation of correct placement by bilateral air entry on chest auscultation was noted.

Correct placement of the devices was confirmed by:

- Adequate chest movement on manual ventilation
- Square wave capnography
- Expired tidal volume of more than 8 ml/kg
- No audible leak from the drain tube with peak airway pressure (PAP) less than 20 cm H _2_O. A leak below 20 cm H _2_O was taken as significant and suggested a malposition
- The gel displacement test, done by placing a blob of gel at the tip of the drain tube (DT) and noting the airway pressure at which it was ejected

The last two tests were specific for group P.

Anaesthesia was maintained with oxygen, nitrous oxide, halothane, and vecuronium.

The outcomes measured were as follows:

- Insertion characteristics of the PLMA or ETT and the nasogastric tube (NGT) via the PLMA and the ETT (NGT was introduced in all cases).
- Easy insertion – insertion at first attempt with no resistance; difficult insertion -insertion with resistance or at second attempt; and failed insertion – insertion not possible.
- Haemodynamic responses(heart rate and mean arterial blood pressure)were recorded before induction; at the time of insertion; 1 and 3, 5 min after insertion of device; after achieving carboperitoneum, and during removal of devices.
- Oxygen saturation (SpO_2_) and end-tidal carbon dioxide (EtCO_2_); at a tidal volume of 8 ml/kg, fraction of inspired oxygen (FiO_2_) 0.33, respiratory rate of 12/min and I/E of 1:2 were recorded.
- The aim was to maintain target SpO_2_(>95%) and EtCO_2_ (<45 mm Hg) by adjusting the FiO_2_, respiratory rate and tidal volume. When SpO_2_ was 94-90% the oxygenation was graded as suboptimal and failed if it was <90%.
- Oropharyngeal seal pressure was determined by closing the expiratory valve at a fixed gas flow of 5 l/min and recording the airway pressure at which equilibrium was reached. The airway pressure was not allowed to exceed 40 cm H_2_O.
- The PAP was recorded when intra-abdominal pressure (IAP) reached 16 mm Hg. For standardisation, IAP was maintained at 12-16 mm Hg.
- Incidences of gastric distension (by surgeon), regurgitation, aspiration, intraoperative and postoperative laryngopharyngeal morbidity were noted.

### Statistical analysis

Data were analysed using INSTAT 3 (GraphPad Software, California, USA). The primary variables studied were oxygenation and adequacy of ventilation. Secondary variables were time to achieve an effective airway, airway interventions required, haemodynamic parameters, cuff leak pressure, and PAP. Sample size of 60, with 30 patients in each group was determined for primary variables (O_2_ saturation and EtCO_2_), using the following information from various previous studies: standard deviations of 5% and 5 mm Hg for the two variables, respectively, were considered statistically significant. If the statistically significant difference in a decrease in oxygen saturation was less than 95% for one of the devices, it was considered to be clinically significant. Sample size was calculated assuming a two-sided test with α = 0.05 and the power of 0.9. Two-sided independent Student’s t tests to analyse continuous data, and Fisher’s exact test for categorical data. *P*<0.05 was considered as significant.

## RESULTS

**Figure 1:**
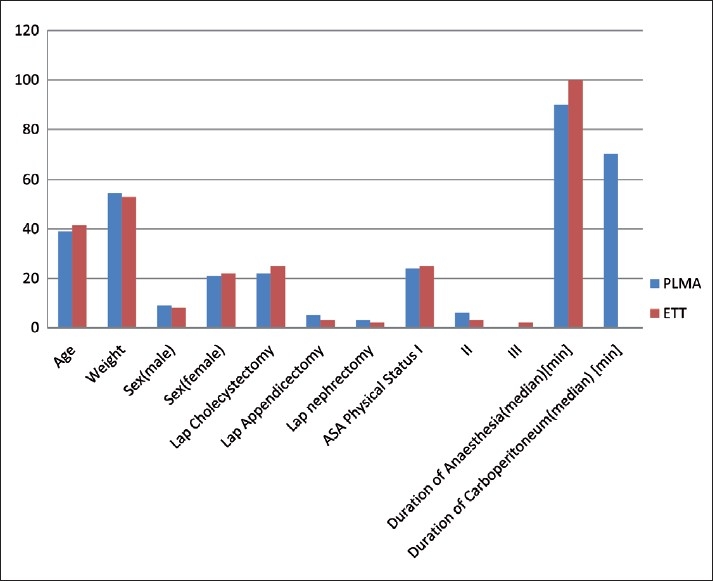
Demographic data and type of procedures done

The surgical procedures, patient characteristics and details of anaesthesia and airway management are shown in Figure 1. Demographic data were comparable in both groups.

Size 3 PLMA placement was attempted in 19 patients, size 4 in 11 patients [Table 1]. Insertion success rate was 86.67% for the first attempt, and two attempts were made in 13.33% patients. Insertion was easy in 23 and difficult in 7 patients. In Group E, the insertion success rate was 83.37% for the first attempt; two attempts were made in 13.33% of patients and third attempt was required in 3.33% patients. There was no failed insertion reported in either group. Mean time (range) taken for successful placement was 15.77 s (12-21 s) and 16.93 s (11-28 s) for PLMA and ETT, respectively.

**Table 1:** Details of airway management

Airway device details	PLMA	ETT	*P* value
Size of device (3/4,7.5/8)	19/11	23/7	
Attempt of insertion (1/2/3/ failed)	26/4/0/0	25/4/1/0	
Time taken for insertion of device, Mean (SD)	15.77 (2.97)	16.93 (4.07)	0.209
Attempts at gastric tube insertion (1/2/3/failed)	27/3/0/0	20/7/3/0	
Time taken for insertion of gastric tube, Mean (SD)	9.77 (2.44)	11.5 (2.28)	0.006
Oropharyngeal seal pressure, Median	35 cm of H_2_O		

Time taken for successful passage of NGT was 9.77 s (6-16 s) and 11.5 s (8-17 s) for P and E groups, respectively.

**Figure 2:**
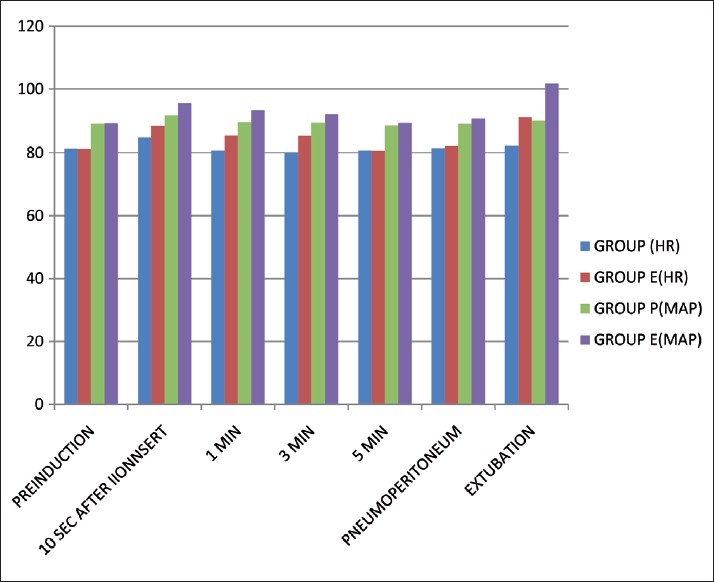
Haemodynamic parameters Statistically significant increase in heart rate and mean blood pressure was observed 10 seconds after intubation and persisted till 3 minutes after intubation and also during extubation in the ETT group. However, statistically significant increase in Proseal-LMA group was seen only 10 seconds after insertion.

On comparing the trends within groups statistically significant (*P*<0.05) increase in heart rate and the mean blood pressure was observed 10 seconds after intubation and persisted till 3 minutes after intubation and during the time of extubation in the ETT group. However, statistically significant (*P*<0.05) increase in the heart rate and mean blood pressure in PLMA group was seen only 10 seconds after insertion [Figure 2].

The EtCO_2_ was comparable in both groups throughout the surgery (*P*>0.05) and did not increase beyond 45 mm Hg.

The PAP in group P showed a statistically significant (*P*<0.05) increase in value after insertion till 6 minutes after pneumoperitoneum was attained, and thereafter it was insignificant.

Both groups maintained oxygen saturation perioperatively except in one patient in the PLMA group where oxygen saturation dropped to 94% (suboptimal oxygenation) after placing patients in the reverse Trendelenburg position. The oxygen saturation returned to normal after the PLMA was repositioned.

Oropharyngeal seal pressure for PLMA group observed was 35 mm Hg (median), with no clinically audible leak throughout the surgery. The PAP, however, did not increase beyond the oropharyngeal seal pressure in the PLMA group [Table 1].

In the present study, coughing after removal of PLMA was seen in 6.67% patients, while it was seen in 3.33% patients in the ETT group. Blood staining of device on removal was seen in 10% patients in group P and in 16.67% patients in group E. Minor trauma to the lip and gums was seen in 1 patient (3.33%) in group E. There was no incidence of intraoperative or postoperative laryngospasm, bronchospasm, in either group. There was no incidence of regurgitation or clinically detectable pulmonary aspiration in either group [Table 2]. In 3 patients, gastric distention was successfully decompressed via NGT suction. Sore throat postoperatively was seen in 10% patients in group P and in 20% patients in group E. After 24 hours, no patient in group P but 2 patients (6.67%) in E group complained of sore throat.

**Table 2:** Laryngopharyngeal morbidity

	PLMA	ETT	*P*
Intraoperative			
1. Leak	1	-	
2. Gastric insufflation	3	-	
3. Regurgitation, aspiration	-	-	
At removal			
1. Coughing	2	1	0.556
2. Blood staining of device	3	5	0.45
3. Trauma to lip, teeth, tongue	4	1	0.17
Postoperative			
1. Vomiting	-	-	
2. Sore throat	3	7	0.171
3. Dysphagia, dysphonia, dysarthia	-	-	

## DISCUSSION

The PLMA is a new entrant to the family of LMA with some added features over the classic LMA.[3] This study was conducted with the aim of comparing PLMA and ETT as a ventilatory device in 60 patients undergoing laparoscopic surgeries. We chose this study because increased intra-abdominal pressure from pneumoperitoneum requires higher airway pressures for adequate pulmonary ventilation, for which the PLMA has proved to be adequate in previous[1,2,4] studies. Although PLMA was easier to insert with higher success rate (86.67%) in the first attempt than the ETT (83.33%), this was not statistically significant. Mean time taken for successful placement was 15.77 s and 16.93 s for groups P and E, respectively. Studies by Cook, Shroff and coworkers (median effective time 15 s) corroborated with our study findings.[4,5] Sharma and coworkers, in their study of 100 and 1,000 PLMA insertions, reported a mean insertion time of 13.51 s and 12 s, respectively.[1,6] This lesser time could be attributed to the fact that their study was conducted by anaesthesiologists who had more experience in working with PLMA.

A NGT was inserted in all patients. The mean insertion time taken to insert NGT through PLMA was significantly less (9.77 s) than via nose (11.5 s) in intubated patients. Similarly, the success rate of NGT in the first attempt was higher (90%) via Proseal than via nasal route in intubated patients (66.67%). These factors may be of clinical relevance in patients with hypertension, head injury, and ischaemic heart disease.

There was minimum haemodynamic stress response with PLMA when compared with endotracheal intubation. These findings are similar to those of previous studies.[1,2,7]

The increase in heart rate during intubation is attributed to sympathetic stimulation during laryngoscopy and the passage of the ETT through the vocal cords.[1,8,9] The PLMA being a supraglottic device does not require laryngoscopy and probably does not evoke a significant sympathetic response. Attenuation of this response may be due to diminished catecholamine release.[10] This could be due to the fact that the PLMA is relatively simple and atraumatic to insert and does not require laryngoscopy.[9]

Following peritoneal insufflation, CO_2_ is absorbed transperitoneally, and the rate at which this occurs depends on gas solubility, perfusion of the peritoneal cavity, and duration of the pneumoperitoneum.[11] Both groups maintained adequate oxygenation and ventilation perioperatively, except in one patient in the PLMA group, where oxygen saturation dropped to 94% (suboptimal oxygenation) after placing the patient in reverse Trendelenburg position. We repositioned the PLMA and oxygen saturation returned to normal thereafter.

Maltby *et al*. and Sharma *et al*. found no statistically significant differences in SpO_2_ or EtCO_2_ between the two groups before or during peritoneal insufflations.[7,11]

However, Sharma and colleagues in a later study noted that although all patients had optimal oxygenation, three patients had EtCO_2_ in excess of 55 mm Hg after CO_2_ insufflation.[6] This was explained by the fact that the airway tube was narrow and the epiglottis downfolded in some patients. The incidence of epiglottic downfolding has been reported to be as high as 31-66%.[12]

The observed oropharyngeal seal pressure for PLMA group was 35 mm Hg (median), with no clinically audible leak throughout the surgery. The PAP did not increase beyond the oropharyngeal seal pressure throughout surgery. This is in accordance with the findings of previous studies.[1,3,6,7]

In three patients, gastric distention was successfully decompressed by suctioning the NGT. There was no incidence of regurgitation or aspiration in either group. Similar results have been reported by others.[1,13,14]

The incidence of sore throat was comparatively more in the intubation group E (20%) than in group P (10%). All patients were administered gargles and steam inhalation. After 24 hours, none of the patients in the Proseal group had sore throat; however, two patients in group E had persistent sore throat till 48 hours. Higgins *et al*. and Shroff *et al*. also found the greatest incidence of sore throat in patients undergoing intubation than in those in whom a PLMA was used.[4,14] The virtual absence of sore throat in PLMA group could be explained by the fact that it is a supraglottic device and mucosal pressures achieved are usually below pharyngeal perfusion pressures.[15]

Although endotracheal intubation is the gold standard in laparoscopic surgeries done under general anaesthesia, the PLMA proved to be an equally effective airway tool in laparoscopic surgeries in terms of adequate oxygenation and ventilation with minimal intraoperative and postoperative complications. The haemodynamic stress response was also minimal with PLMA when compared to endotracheal intubation. It provided equally effective pulmonary ventilation despite high airway pressures without significant gastric distention, aspiration, and regurgitation.

## CONCLUSION

Hence, we conclude that the PLMA proved to be a suitable and safe alternative to ETT for airway management in elective fasted, adult patients undergoing laparoscopic surgeries.

## References

[1] Sharma B, Sahai C, Bhattacharya A, Kumar VP, Sood J (2003). ProSeal laryngeal mask airway: A study of 100 consecutive cases of laparoscopic surgery. Indian J Anaesth.

[2] Misra MN, Ramamurthy B (2008). The Pro-Seal LMAtm and the tracheal tube: A comparison of events at insertion of the airway device. Internet J Anesthesiol.

[3] Brain AI, Verghese C, Strube PJ (2000). The LMA ‘ProSeal’ - a laryngeal mask with an oesophageal vent. Br J Anaesth.

[4] Shroff P, Surekha K (2006). Randomized comparative study between the proseal laryngeal mask airway and the endotracheal tube for laparoscopic surgery. Internet J Anesthesiol.

[5] Cook TM, Nolan JP, Verghese C, Strube PJ, Lees M, Millar JM (2002). A randomized crossover comparison of the proseal with the classic laryngeal mask airway in unparalysed anaesthetized patients. Br J Anaesth.

[6] Sharma B, Sood J, Sahai C, Kumara VP (2008). Efficacy and safety performance of proseal laryngeal mask airway in laparoscopic surgery: Experience of 1000 cases. Indian J Anaesth.

[7] Maltby JR, Beriault MT, Watson NC, Liepert DJ, Fick GH (2002). The LMA proseal is an effective alternative to tracheal intubation for laparoscopic cholecystectomy. Can J Anaesth.

[8] Fujii Y, Tanaka H, Toyooha H (1997). Circulatory responses to laryngeal mask airway insertion or tracheal intubation in normotensive and hypertensive patients. Can J Anaesth.

[9] Evans NR, Gardner SV, James MF, King JA, Roux P, Bennett P (2002). The proseal laryngeal mask: Results of a descriptive trial with experience of 300 cases. Br J Anaesth.

[10] Lamb K, James MF, Janicki PK (1992). Laryngeal mask airway for intraocular surgery, effects on intraocular pressure and stress responses. Br J Anaesth.

[11] Piper J, Fenn WO, Rahn M (1965). Physiological equilibria of gas cavities in the body. Handbook of Physiology. Section 3: Respiration.

[12] Brimacombe J (2003). The proseal laryngeal mask airway: An easier and safer approach to tracheal tube/ laryngeal mask exchange. Anaesthesia.

[13] Maltby JR, Beriault MT, Watson NC, Liepert DJ, Fick GH (2003). LMA-classic and LMA-proseal are effective alternative to endotracheal intubation for gynecologic laparoscopy. Can J Anaesth.

[14] Higgins PP, Chung F, Mezei G (2002). Postoperative sore throat after ambulatory surgery. Br J Anaesth.

[15] Rabey PG, Murphy PJ, Langton JA, Barker P, Rowbotham DJ (1992). The effect of the laryngeal mask airway on lower oesophageal sphincter pressure in patients during general anaesthesia. Br J Anaesth.

